# Barriers to clinical research in children with inflammatory bowel disease: The patients' perspective

**DOI:** 10.1371/journal.pone.0206965

**Published:** 2018-11-08

**Authors:** Wael El-Matary, Vini Deora, Kanika Grover

**Affiliations:** Section of Pediatric Gastroenterology, Department of Pediatrics and Child Health, University of Manitoba, Winnipeg, Manitoba, Canada; McMaster University Michael G DeGroote School of Medicine, CANADA

## Abstract

**Background:**

The aim of this study was to determine the proportion of patients’ caregivers willing to participate in clinical research and examine the possible barriers against recruitment to clinical research in children with inflammatory bowel disease.

**Methods:**

In a cross-sectional study, caregivers and children with inflammatory bowel disease were surveyed via a questionnaire that addressed parents’ willingness to participate in clinical studies and factors influencing their willingness to participate.

**Results:**

A total of 118 caregivers to children with inflammatory bowel disease [median age 14.5, IQR: 12.0–15.8 years, 60 boys, 61 (52%) with Crohn’s disease] who were followed for a median duration of 1.73 years (IQR 0.4–3.6 years) completed the survey. One hundred and four (88.2%) caregivers answered “Definitely” or “Probably” to participate in clinical research while 14 (11.8%) were “Neutral” or “Probably” unwilling to participate (P<0.001). Patients were less likely to participate in clinical research if they had longer disease duration (P = 0.019), or were in clinical relapse (P = 0.03). Parents’ education, income, age of children at diagnosis, money incentive, disease relapse and medications at the time of the survey did not have any significant effect on willingness to participate.

**Conclusions:**

The majority of children with inflammatory bowel disease and their caregivers are willing to participate in clinical research.

## Introduction

Inflammatory bowel disease (IBD) is a group of chronic immune system disorders characterized by remissions and relapses. Inflammatory bowel disease encompasses two related but distinct disorders: Crohn’s disease (CD) and ulcerative colitis (UC). CD is a chronic, idiopathic, transmural, patchy, inflammation of one or more segments of the gastrointestinal tract. UC is a chronic, idiopathic, diffuse mucosal inflammation of the colon [1, 2]. For patients with no clear features of either UC or CD, the term IBD-unclassified (IBD-U) could be employed.

The peak incidence of IBD is between the ages of 10 and 30 years. A recent study showed that 8–10% of all patients with IBD usually present in childhood and adolescence [3]. In Manitoba, we have recently shown that the sex- and age-adjusted incidence of pediatric CD has significantly increased from 1.2/100,000 in 1978 to 4.68/100,000 in 2007. For UC, the incidence has increased from 0.47/100,000 in 1978 to 1.64/100,000 in 2007. During the same time period, the prevalence of CD has significantly increased from 3.1 to 18.9/100,000 and from 0.7 to 12.7/100,000 for UC [4].

Scarcity of evidence-based knowledge in many aspects of IBD is a major challenge that could be related to several barriers. Those barriers include research funding difficulties, research-design related factors (such as randomized controlled studies versus observational studies), and physicians and health workers-related factors. For example, patients may have lower threshold to consent for research conducted by their treating physicians versus physicians and health care workers who are not involved in their care [5,6]. In a survey among North American Gastroenterologists examining barriers against clinical research, uncompensated research costs and a lack of specialized support were the most common barriers to research [6].

One major barrier to clinical research is the reduced willingness of patients to participate in clinical research [5,6]. Data on barriers for recruitment are limited, especially in the pediatric age group. The aim of this study was to determine the proportion of patients’ caregivers willing to participate in clinical research and examine possible barriers against clinical research in children with IBD from the perspective of patients and their caregivers.

## Subjects and methods

### Study design and participants

In a cross-sectional analysis, patients (< 18 years) with an established diagnosis of IBD following the Porto diagnostic criteria by the European and North American Society of Pediatric Gastroenterology, Hepatology and Nutrition (ESPGHAN & NASPGHAN) [7] and have been followed by the Section of Pediatric Gastroenterology at the Winnipeg Children’s Hospital and their caregivers were invited to answer a survey after obtaining all necessary consents. A hundred and twenty patients with IBD are followed in our IBD clinic. The study was conducted between December 2016 and July 2017.

### Study procedures

The demographic data from the list of pediatric IBD patients who were followed by the Section of Pediatric Gastroenterology were examined. Potential participants were contacted by telephone by the study co-coordinator. The study was explained over the phone and a verbal consent from patients and caregivers was obtained. Once the consent was obtained, a phone survey with patients (when appropriate, depending on the patients’ age) or their caregivers was conducted.

### Study tool

In addition to the main question addressing patient’s willingness to participate in research studies, the survey had 25 questions divided among the 3 main domains:

- Research-related factors: such as research design, frequency of visits and nature of required biological samples
- Patient-related factors: such as family income and parents’ education
- Disease-related factors: such as subtype and duration of the disease and medications

A 5-point Likert scale (definitely, probably, neutral, probably not, definitely not) was used to capture the response information for willingness to participate.

The questions were adopted from surveys employed in previous studies [5–7] and piloted before formal recruitment. Ten children and young adolescents representing different age groups were randomly chosen and were asked to complete the survey. We observed them and their caregivers during answering the survey and we asked for their feedback. The questionnaire was then modified in view of the suggestions that were received.

### Data collection

Clinical data on disease subtype, duration, disease distribution, clinical disease activity at the time of the survey and IBD-related surgery and medications were collected from medical records. The postal codes of the participants’ residences were collected as an indicator for their socioeconomic status (SES).

The primary outcome was the proportion of caregivers who are willing to participate in clinical research. Secondary outcomes included factors affecting willingness of patients/caregivers to participate in clinical research including research-related factors such as study design such as randomized controlled studies versus cohort studies (both were explained by the research assistant) and type of biological samples to be obtained for research. Other factors included patient and disease-related factors such as demographics and disease status.

### Statistical analysis

Data analysis was performed using Stata 9.1 (TM) (Data Analysis and Statistical Software, College Station, Texas, USA). Univariate summaries (median and interquartile range (IQR)) were obtained for the continuous variables (i.e. age), while frequency distributions were provided for categorical variables (e.g. gender, level of education attained by parents) along with the 95% confidence intervals (CIs) for the means and proportions. Proportions were compared using the *Chi-square test* or *Fisher’s* exact test where appropriate, while a two-sided *Student’s t-test* was used to compare continuous variables. Univariate logistic regression analysis was used to examine the possible effect of variables examined such as educational levels attained by parents and family income. Model assumptions were assessed using residual diagnostic techniques. A P value of 0.05 was used for defining the level of significance.

### Ethical considerations

The study protocol was approved by the Health Research Ethics Board of The University of Manitoba.

## Results

A total of 118 (98.3%) out of 120 caregivers of children (age < 18 years) with IBD who were approached completed the survey (Median age 14.46, IQR: 12.00–15.75 years, 60 boys, 61 CD). 110 (92%) participants were over the age of 7 years. Patients were followed for a median duration of 1.73 years (IQR 0.4–3.6 years). Patients’ demographics are summarized in Table 1. 14 (12%) participants had at least one sibling diagnosed with IBD.

**Table 1:** Demographic characteristics of participants. *As defined by clinical disease activity indices.

**Age (years**	
Mean	13.54± 3.23
Median	14.46
Interquartile Range	12.00–15.75
**Gender**	
Male	60 (51%)
Female	58 (49%)
**Mother's Education**	
Bachelor's Degree	58 (49%)
No Degree	59 (50%)
Don't know	1 (1%)
**Spouse's Education**	
Bachelor's Degree	55 (46%)
No Degree	54 (45%)
Don't know	1 (1%)
No spouse	8 (7%)
**Family Income**	
<$31,000	4 (3%)
>$31,000	74 (62%)
Prefer not to answer	40 (35%)
**Disease type**	
Crohn's Disease	61 (51%)
Ulcerative Colitis	56 (48%)
Inflammatory Bowel Disease-Unclassified	1 (1%)
Currently in Relapse*	11 (9%)
**Previous Surgery**	7 (6%)
**On Biologic (Infliximab or Adalimumab)**	54 (45%)
**On an Immunosuppressant (Methotrexate or Azathioprine)**	74 (62%)
* As defined by clinical disease activity indices	

One hundred and four (88.2%) caregivers were “Definitely” or “Probably” willing to participate in clinical research while 14 (11.8%) were “Neutral” or”Probably” unwilling to participate (P<0.001). Responses are summarized in Table 2.

**Table 2:** Willingness to participate in clinical research of children with IBD.

**Willingness to participate**	**No. of patients**	**%**
Definitely	92	78
Probably	12	10.2
Neutral	11	9.3
Probably not	3	2.5
Definitely Not	0	0.0
Total	118	100

Seventy four (62.7%) participants previously participated in at least one study and 21 (17.8%) previously declined participation in one or more research studies. The majority of participants [71 (60.2%)] patients thought that a monthly clinic visit would not make any difference in encouraging participation in clinical research.

Ninety participants (76.3%) agreed to participate in studies that include obtaining “two small” blood samples, 88 (74.6%) agreed to give one urine sample, and 71 (60.2%) agreed to give a stool sample. On the other hand, 84 (71.2%) patients would decline participating in studies that include obtaining endoscopy biopsy samples.

Fifty eight (49.2%) participants thought that money ($100) would encourage participation in clinical research (P = 0.8).

Patients were less likely to participate in clinical research if they had longer disease duration (P = 0.019), or were in clinical relapse at the time of participation (P = 0.03). Patients were also less likely to participate if they were asked to provide tissue biopsy samples (P = 0.05). Other biological samples such as blood samples (P = 0.7), urine (P = 0.1) or stool (P = 0.8) samples did not seems to make any difference on patients’ willingness to participate. Nature of research studies such as randomized controlled studies (P = 0.2) or open labelled studies (P = 0.3), family history of IBD (P = 0.4), parents’ education (P = 0.8), family income of over $ 31,000.00 per annum (P = 0.5), money incentive (P = 0.8), and medications, such as immunosuppressive or biological medications, at the time of the survey did not have any significant effect on willingness to participate.

## Discussion

The variety of barriers against recruitment for clinical research studies in children with inflammatory bowel disease (IBD) needs to be identified and examined in order to work towards resolving these barriers and encourage recruitment. In our study, the majority of children and their caregivers were willing to participate in clinical research, especially if they had shorter disease duration or were in clinical remission at the time of the survey.

Studies examining barriers to recruitment in children with chronic diseases including IBD are scarce. In a cross-sectional study, Hoberman and colleagues surveyed parents previously asked to provide consent for their child’s participation in the trial about factors that influenced their decision. Having graduated from college and private health insurance were associated with lower likelihood of providing consent. Parents who perceived the study as having a low degree of risk, resulting in greater benefit to their child and other children, causing no or minor interference with the standard care or who perceived the researcher as professional were significantly more likely to consent to participate [8]. In our study, parents’ education did not seem to have a major association with willingness to participate in clinical research. A major limitation of the study by Hoberman *et al* was the low participation rate of the survey as only 44% of those who were invited to participate actually answered the survey questions.

In comparison to our study, invasive procedures such as colonoscopies or sigmoidoscopies did not have a positive influence on willingness to participate in research studies for adults with IBD in a study that surveyed 200 patients with IBD [9]. Other factors that interfered with willingness to participate included the need for frequent physician visits and the blinding design. Male gender, compensation, and active disease at the time of survey were associated with higher willingness to participate [9]. A telephone interview with patients with systemic lupus erythematosus who accepted or refused to participate in clinical trials indicated the presence of several factors that affected the decision-making process of participation. Those factors included health status at the time of participation, study design, physician involvement, altruism, personal benefit and incentives. Good health status, encouragement from one’s physician and personal desire to learn and contribute stimulated participation [10]. The gender of participants is another potential factor that may affect willingness to participate in clinical research. Ding et al. explored this factor in a multi-center study to examine the willingness of patients with cardiovascular disease to participate in clinical trials. Women were less willing to participate as they perceived greater risks of chronic disease-related harm [11]. We did not encounter the same finding in our study as parents’ views for their children to participate in clinical research may be different from their willingness for their own participation i.e. Parents may have a lower threshold to participate in clinical research as they feel less anxious compared to their feelings towards their children’s participation.

Although ethically controversial, paying participants as a motivator for participation in clinical research was explored among other factors in a study by Halpern et al [12]. Willingness to participate decreased by lower payment level, higher risks of adverse events, and higher risks for being assigned to placebo. Higher payments did not alter participants’ reaction to the examined risk factors such as adverse events, suggesting that higher payments did not alter participants’ perception of risks. Interestingly, in this study, wealthier participants were more likely to state that payment was important in their participation decision [12]. In our study, paying money as an incentive for participation did not seem to be associated with a higher willingness to participate.

Our study is unique and novel. This is the first study examining barriers to clinical research among parents and children with IBD. In addition, the study has an excellent response rate to the invitation to complete the survey reaching up to 98.3%. This may reflect a high level of motivation of both patients’ caregivers and our research team.

On the other hand, our study is limited by using a non-validated survey tool. However, there is no validated tool in this context.

Although the majority of our participants were willing to participate, actual consent for participation in clinical research may vary. Nonetheless, our study highlights some factors and difficulties that should be taken into consideration when researchers plan for studies for children with IBD. Disease relapse may not be the best time for approaching participants. Researchers should be aware that they may not be able to obtain endoscopy biopsy and stool research samples in up to 70% and 40% of children with IBD respectively. This could be proven valuable in sample size calculation. On the other hand, patients seem to be willing to donate blood samples over stool samples. This might be explained by the easiness to collect blood over stools and the fact that adolescents seem to feel disgusted by the process of stool collection.

## Conclusions

The majority of caregivers of children with IBD, especially those with Crohn’s disease, are willing to participate in clinical research. Patients are less likely to consent for clinical research if they have longer disease duration and were approached at the time of clinical relapse. Future research studies examining strategies to change the attitude of patients’ caregivers towards certain barriers such as collection of tissue sample biopsies are warranted.

## Supporting information

S1 File (XLS)

S2 File (DOC)
