# Supplementary material for: Barriers to clinical research in children with inflammatory bowel disease: The patients' perspective
Source: PLoS One. 2018 Nov 8;13(11):e0206965. doi: 10.1371/journal.pone.0206965 (PMC6224089; doi:10.1371/journal.pone.0206965)
Supplement: S2 File — (DOC) [file pone.0206965.s002.doc]

**Appendix 1**

ID

age Gender M F

Postal code

1. **Are you willing to participate (allow your child) to participate in a research study?**
2. Definitely
3. Probably
4. Neutral
5. Probably not
6. Definitely not
7. **Are you currently or did you previously participated in research?**
8. Yes 2. No 3. Don’t know
9. **Did you decline to participate in research in the past?**
10. Yes 2. No 3. Don’t know
11. **Which of the following factors would encourage your/your child participation in a research study?**
12. Monthly visits to your physicians
13. Yes b) No c) Does not make any difference c) Don’t know
14. Same physician each visit
15. Yes b) No c) Does not make any difference c) Don’t know
16. Blood samples (2 small samples)

a) Yes b) No c) Does not make any difference c) Don’t know

1. Urine samples

a) Yes b) No c) Does not make any difference c) Don’t know

1. Stool samples

a) Yes b) No c) Does not make any difference c) Don’t know

5) Tissue sample in endoscopy/colonoscopy

a) Yes b) No c) Does not make any difference c) Don’t know

1. Randomization (will be explained)
2. Yes b) No c) Does not make any difference c) Don’t know
3. Open label study (will be explained)

a) Yes b) No c) Does not make any difference c) Don’t know

8) $100 for participation

a) Yes b) No c) Does not make any difference c) Don’t know

9) $ 200 for participation

a) Yes b) No c) Does not make any difference c) Don’t know

1. **Family income per year**
2. < $ 75,000 2) > $ 75,000 3) don’t want to answer
3. **Parents together**

1) Yes 2) No

1. **Single parent**

1) Yes 2) No

1. **Caregiver highest degree of education**
2. Father: a) Bachelors or above b) Less than bachelors
3. Mother a) Bachelors or above b) Less than bachelors
4. **Disease-related information (to be completed from health records by RA)**
5. Disease type
6. Date of diagnosis
7. Duration of disease
8. Disease distribution
9. IBD-related surgery Yes No
10. Currently in relapse Yes No
11. Patient is on immunomodulator Yes No
12. Patient is on biologic Yes No
